# Spatial distribution of vitamin A rich foods intake and associated factors among children aged 6–23 months in Ethiopia: spatial and multilevel analysis of 2019 Ethiopian mini demographic and health survey

**DOI:** 10.1186/s40795-022-00573-0

**Published:** 2022-08-11

**Authors:** Addisalem Workie Demsash, Alex Ayenew Chereka, Sisay Yitayih Kassie, Dereje Oljira Donacho, Habtamu Setegn Ngusie, Masresha Derese Tegegne, Mequannent Sharew Melaku, Sisay Maru Wubante, Mekonnen Kenate Hunde

**Affiliations:** 1 Health Informatics Department, College of Health Science, Mattu University, Mettu, Ethiopia; 2 Health Informatics Department, Institute of Public Health, University of Gondar, Gondar, Ethiopia; 3 Lifelong Learning and Community Development Department, College of Education and Behavioral Scieence , Mattu University, Mettu, Ethiopia

**Keywords:** Vitamin A rich foods, Intake, Children, Spatial distribution, Multilevel, Ethiopia

## Abstract

**Background:**

Inadequate micronutrients in the diet and vitamin A deficiency are worldwide public health problems. In developing regions, many preschool children are undernourished, become blind every year and died before the age of 23 months. This study was aimed to explore the spatial distribution of vitamin A rich foods intake among children aged 6–23 months and identify associated factors in Ethiopia.

**Methods:**

Ethiopian Mini Demographic and Health Survey 2019 dataset with a total 1407 children aged 6–23 months was used. Data management and processing were done using STATA version 15 software and Microsoft Office Excel. ArcMap version 10.7 software was used for mapping and spatial visualization of the distribution. Spatial scan statistics was performed using SaTScan version 9.5 software for Bernoulli-based model. Multilevel mixed effect logistic regression model was employed to identify associated factors.

**Results:**

Overall, 38.99% (95% CI: 36.46–41.62) of children aged 6– 23 months took vitamin A rich foods. Poor intake of vitamin A rich foods was significantly clustered Dire Dawa city, Somali and Harari regions of Ethiopia. Children aged 6–23 months lived in the primary cluster were 70% (RR = 1.70, *P*-value < 0.001) more likely to intake vitamin A rich foods than children lived outside the window. In the multilevel mixed effect logistic regression analysis, Primary educational status (AOR:1.42, 95% CI: 1.05, 1.93) and higher educational status (AOR:3.0, 95% CI: 1.59, 5.65) of mother, Dire Dawa (AOR:0.49, 95% CI: 0.22, 1.12) city, Afar (AOR: 0.16, 95% CI: 0.07, 0.36), Amhara (AOR: 0.37, 95% CI: 0.19, 0.71) and Somali (AOR: 0.02, 95% CI: 0.003, 0.08) regions of Ethiopia, children aged 13–23 months (AOR: 1.80, 95% CI: 1.28, 2.36), Mothers’ exposure to media (AOR: 1.41, 95% CI: 1.04, 1.92) were statistically significant factors for vitamin A rich foods intake among children aged 6–23 months.

**Conclusions:**

Only 4 out of ten children took vitamin A rich foods which is too low compared to the national target and significantly clustered in Ethiopia. Mother’s educational status, Region, Child age and Mother’s media exposure are significant factors vitamin A rich foods intake. Stakeholders should strengthen mothers’ education status, creating awareness for mothers on child feeding and using locally available natural resource to produce vitamin A rich foods.

## Background

Micronutrients are essentially needed for optimal health [1, 2] and play a critical role in cellular and humoral immune responses, cellular signaling and function, child growth and development, and learning and cognitive functions [3, 4]. The World Health Organization (WHO) recommended appropriate plan-based complementary foods, and animal products such as meat, poultry, fish, or eggs to ensure the number of nutrient requirements for children [5].

Micronutrients (MNs) deficiency among children is a worldwide public health problem, and it is a predisposing for any disease associated with children [6, 7]. Even though MNs are only needed in fewer amounts, their absence and inadequacy in diet negatively affect children’s survival and development [8], and leads to deliberate causes of anemia, stunting, wasting, weak immunity, and delay in cognitive development [9]. Vitamin A deficiency (VAD) in children is a critical factor for children’s death due to measles, diarrhea, malaria, and infectious disease [10].

Globally, MNs affect social and economic development, and it accounts for 2.655 million deaths of under-five children occurred in 2015 [11]. WHO estimates that 5.2 million preschool children are affected by VAD [12]. Nearly, 250 million preschool children are vulnerable to VAD and one-fourth million children become blind. As a result, half of them die before 23 months [13].

In developing countries, MNs deficiency is a common public health problem for children [14].South Asia and sub-Saharan Africa are the home of chronically undernourished children than elsewhere in the world [15, 16]. Over 200 million African children suffer from malnutrition and fail to reach their cognitive potential stages [17]. Different literatures reported VAD ranging from 8.5% to 79% in Africa [18, 19]. According to a previous study in different parts of Africa, intake of vitamin A and vitamin A-rich foods in suboptimal( 52%) in Ghana [19]. The demographic and health survey (DHS) report of Malawi in 2015 [20] and Uganda in 2016 [21] reveals 79.1% and 66.5% of vitamin A-rich food intake among children respectively.

In Ethiopia, VAD continued to be a major public health problem among children and the problem varies from region to region [22]. Only 8.5% of the children had the recommended minimum dietary diversity [23]. According to the Ethiopian 2016 DHS report, 38% of children aged 6–23 months had an intake of vitamin A-rich foods within 24 h [24] which is 1% lower than the 2019 Ethiopian Mini Demographic Health Survey (EMDHS) report [25]. Mother’s educational status and their range of age, Family’s wealth index, age and sex of children [23], household exposure to media, and household income, children’s weight and size at birth [18, 26] were major factors affecting vitamin A-rich foods intake among children aged from 6–23 months.

Though different primary studies have been conducted to assess the nutritional status of children in Ethiopia, none of the previous studies explored the spatial distribution of vitamin A rich foods intake is inadequate, except on poor consumption of vitamin A-rich foods [27]. Hence, Exploring the spatial patterns and distributions of vitamin A-rich foods intake in the regions of Ethiopia is used for further understanding of where the intake of vitamin A-rich foods among children occurs in a specific location and helps for better nutritional interventions. Therefore, this study was aimed to explore the spatial distribution of vitamin A rich foods intake and identify associated factors among children aged 6–23 months in Ethiopia.

## Methods

### Study design and setting

A repeated cross-sectional study design was conducted in Ethiopia. Ethiopia is located in the Horn of Africa and bordered by Eritrea to the north, Djibouti, and Somalia to the east, Sudan and South Sudan to the west, and Kenya to the South. Ethiopia is home to about 13 million children under 5 years of age, approximately 16% of the total population [28]. Ethiopia has 9 Regional states with two administrative cities. These are subdivided into different administrative Woredas and further divided into the smallest administrative units in the country called Kebele.

### Data source

For this study, the 2019 Ethiopian Mini Demographic and Health Survey (EMDHS) dataset was used, the second EMDHS and the fifth DHS implemented in Ethiopia. The survey was conducted by Ethiopian Public Health Institute (EPHI) in collaboration with the Central Statistical Agency (CSA). The 2019 EMDHS generates data for measuring the progress of the health sector goals set under the Growth and Transformation Plan (GTP), which is closely aligned with the Sustainable Development Goals (SDG) [25]. The survey was conducted from March 21, 2019, to June 28, 2019. Shapefiles were downloaded from the Africa open data website (https://www.africaopendata.org).

### Sampling procedures and populations

The 2019 EMDHS was conducted by the CSA, and a complete list of the 149,093 enumeration areas (EAs), covering an average of 131 households, was created for the 2019 Ethiopia Population and Housing Census (EPHC). A two-stage stratified cluster sampling was used. Each region was stratified into urban and rural areas, yielding 21 sampling strata.

At the 1^st^ stage of selection, a total of 305 EAs (93 in urban, 212 in rural) were selected independently with a probability proportion to each EAs. At 2^nd^ stage of selection, a fixed number of 30 households/cluster were selected with an equal probability systematic selection from the newly created household listing [25]. The detailed sampling procedures were presented in 2019 EMDHS report from the measure DHS website (https://www.dhsprogram.com). In this study, all living children aged 6–23 months were the source population, and all sampled living children aged 6–23 months living with their mother were the study population. Zero coordinates and clusters which had no a proportions of vitamin A rich foods intake were considered as an exclusion criteria.

### Study variables and their measurements

#### Dependent variable

Vitamin A rich foods intake among children aged 6–23 months is the dependent variable of the study which was determined by respondents’ reports and assessment of vitamin A rich foods [29, 30]. Vitamin A rich foods were measured by the seven food items such as 1. Eggs, 2. Meat (beef, pork, lamb, chicken), 3. pumpkin, carrots, and squash (yellow or orange inside), 4. fish or shellfish, 5. Any dark green leafy vegetables, 6. Liver, heart, and other organs 7. Mangoes, papayas, other Vitamin A fruits. Accordingly, if the respondent reported that the child had took at least one of those vitamin A rich foods item was considered as "Yes", otherwise "No" [8].

#### Independent variables

Potential predictor variables such as Sex of children, child Age (Month), baby postnatal checkups, Educational status of mother, Mother’s Age (Year), Religion of mother, Current Marital and Pregnancy status of mother, ANC visit and Place of delivery, and Wealth status, Mother exposure to media, Sex of household head were individual level independent variables. Whereas, Place of residency, and Region of mothers were taken as community level predictor variables for this study.

#### Media exposure

Recently, infant and child feeding practice is related to media (radio, Television) spots, and access to media may help to hear nutritional information, or messages [31]. Therefore, mother who access to media offer a diversified diet to their children, and so considered mother had media exposure. Otherwise, mothers had not media exposure.

#### ANC visit

In this study, if the child’s mother had visited the health facility at least four times for ANC service during their pregnancy was considered as children’s mother had adequate ANC visit. Otherwise, inadequate ANC visit [32].

### Data management and processing

Data cleaning, labeling, and processing was done using STATA version 15 software and Microsoft Office Excel. To yield accurate parameters estimation, and to handle representativeness of the survey, sampling weight was done. The descriptive analysis results were presented in table and text narrations.

### Spatial data analysis

#### Global spatial autocorrelation and hot spot analysis

ArcMap version 10.7 software was used for spatial autocorrelation and detection of hot spot areas analysis. Global spatial autocorrelation (Global Moran’s I) statistic measure was used to assess whether vitamin A-rich foods intake among children was dispersed, clustered, or randomly distributed in Ethiopia [33]. Moran’s I values close to minus one (-1), close to plus one (+ 1), and if it is zero (0) indicate a dispersed, clustered pattern and random distribution vitamin A rich foods intake among children aged from 6–23 months respectively [34, 35]. A statistically significant Moran’s 1 value (*P* value, 0.05) had a chance to reject null hypothesis which indicate the presence of spatial autocorrelation. Vitamin A rich foods intake among children with either hot spot or cold spot values for the spatial clusters are determined by the z scores and significant *p*-values of hot spot analysis [36, 37].

#### Spatial interpolation

Vitamin A rich foods intake among children aged 6–23 months in the unsampled areas of the country were predicted by using the spatial interpolation technique. To predict Vitamin A rich foods intake among children aged 6–23 months in the unsampled areas, current vitamin A rich foods intake among children aged 6–23 months on sampled areas was used as an input. To minimize prediction uncertainty and filter out measurement errors, Ordinary Kriging Gaussian interpolation technique was employed. Based on the input data at each locations, semi variogram model was constructed, and used to define the weight that furtherly determine the prediction of new values at unsampled areas. As a result, a new simulated semi variogram model was generated [38, 39].

#### Spatial scan statistics

The Sat Scan version 9.5 software was used for the local cluster detection analysis [40]. We employed purely spatial Bernoulli-based model scan statistics to determine the geographical locations of statistically significant clusters with high rate of vitamin A-rich foods intake among children [41]. Those children who intake vitamin A rich foods were taken as cases and those who didn’t intake foods rich in vitamin A were taken as controls to fit the Bernoulli model for the scanning window that moves across the study area. The scanning window that moves outside the study area was clipped. The default maximum spatial cluster size of < 50% of the population was used as an upper limit, allowing both small and large clusters to be detected and ignored clusters that contained more than the maximum limit with the circular shape of the window. For each potential cluster, a log-likelihood ratio test statistic (LLR) was used to determine if the number of observed cases within the potential cluster was significantly higher than expected or not. The circle with the maximum likelihood ratio test statistic was defined as the primary cluster, then compared with the overall distribution of maximum values. The significant clusters were identified according to their *p* values and ranked based on their likelihood ratio (LLR) test based on the 999 Monte Carlo replications [42].

#### Multilevel logistic regression analysis

The assumption of independence among observations was violated due to the hierarchical and clustering nature of EDHS data. This implies a need to consider variability between-cluster by using advanced models since there are concerns which could not be addressed by basic logistic regression models. Four models were considered in the multilevel logistic regression: Model A = empty without out explanatory variable which examines vitamin A rich food intake without the explanatory variables that specified only the random intercept and the overall variance of vitamin A rich food intake among clusters, Model B = individual level variable, Model C = community level variables, Model D = both individual and community level factors. Measurement of variation and correlation also were determined using variance and interclass correlation (ICC). As a result, 28.4% of variance and 30.2% of ICC’s values confirmed that there were significant variations and correlations on vitamin A rich food intake among children aged 6–23 months in the country. Hence, multilevel mixed effect logistic regression analysis was fitted to assess both individual and community level variables. Finally, the model fitted was selected based on Akaike’s Information Criteria (AIC) and Log Likelihood Ratio (LLR). Variables having *p* value up to 0.2 in the bi-variable analysis were selected to fit the model in the multi variable analysis. Finally, *p*-value less than 0.05 in the multivariable model of mixed-effects logistic regression was used to select variables which had statistically significant association with vitamin A rich food intake.

### Ethics approval

Ethical approval and consents from study participant were not necessary for this study because the study was based on secondary data source which is publicly available from the Measure DHS program website (https://www.dhsprogram.com). After a clear working plan, and description on how to use the DHS data was written, request was sent to the Measure DHS program to download and used for this study. As a result, we obtained permission to access the EMDHS 2019 data through (https://www.dhsprogram.com/Date/terms-of-use.cfm) for statistical analysis and reporting. There are no attributes that uniquely identify individuals or household addresses in the data files. The geographic coordinate files are randomly displaced within a large geographic area, and it is only for EAs as a whole. As a result, specific ERs, individuals and households cannot be identified uniquely.

## Result

### Sociodemographic characteristics

From the EMDHS-2019 dataset, a total of 1407weighted sampled children aged 6– 23 months were included for this study. More than half (52.4%) of the children were male, and six out of ten (59.9%) children were between the age of 13 and 23 months. Majorities (86.7%) of the children had not postnatal checkups within 2 months. Nearly one third (32.5%) of mothers were Muslim religious flower. About 38.1% of mothers were from Oromia regional state of Ethiopia.

The majorities such as 95.5% and 72.3% of mothers were married and rural resident respectively. About 86.5% of household heads were male. Six hundred nineteen (45%) of mothers had no formal education and five hundred ninety-five (42.3%) of family were under poor wealth status. Around half (49.4%) of mothers were between 25–34 years of age(Table 1).

**Table 1:** Socio-demographic characteristics of children aged from 6–23 months and Mothers, 2019 EMDHS

Variable	Category	Frequency (n)	Percent (%)
Educational status of mother/caregiver	No formal education	633	45.0
	Primary	536	41.7
	Secondary	115	8.2
	Higher	73	5.2
Region	Tigray	93	6.6
	Afar	19	1.4
	Amhara	310	22.1
	Oromia	537	38.1
	Somali	86	6.1
	Benishangul	16	1.1
	SNNPR	286	20.3
	Gambela	7	0.5
	Harari	4	0.3
	Addis Adaba	42	3.0
	Dire Dawa	8	0.5
Mothers’/caregivers’ age (year)	15–24	458	32.5
	25–34	695	49.4
	> = 35	254	18.0
Sex of household head	Male	1217	86.5
	Female	190	13.5
Family’s wealth index	Poor	595	42.3
	Middle	268	19.0
	Rich	544	38.6
Mother religion	Orthodox	508	36.1
	Catholic	6	0.5
	Protestant	407	28.9
	Muslim	457	32.5
	Traditional, and other	29	2.1
Mothers place of residency	Urban	390	27.7
	Rural	1017	72.3
Sex of children	Male	738	52.4
	Female	670	47.6
The current age of children (months)	6–12	564	40.1
	13–23	843	59.9
Baby postnatal check up	No	1219	86.7
	Yes	188	13.3
Mothers’ current marital status	Married	1344	95.5
	Lived as single	63	4.5
ANC Visit during pregnancy	Inadequate ANC visits	788	56.0
	Adequate ANC visits	619	44.0
Household has radio	Yes	351	24.9
	No	1056	75.1
Household has Television	Yes	235	16.7
	No	1172	83.3
Place of delivery	Home	665	47.3
	Health facility	742	52.7
Mother’s current pregnancy status	Non-pregnant	1326	94.2
	Pregnant	81	5.8

### Vitamin A rich foods intake among children aged 6– 23 months in Ethiopia

Overall, 38.99% (95% CI: 36.46–41.62) of children aged 6–23 months had an intake of food rich in vitamin A in the last 24 h in Ethiopia. Intake of eggs, any dark fruit leafy vegetables and mangoes, papayas, other Vitamin A fruits were the most taken food items which 18.2%, 12%, 11.5% of children intake them respectively, whereas fish or shellfish, meat (beef, pork, lamb, chicken), and liver, heart, and other organs were the least intake food items which 1.8%, 4.8% and 3.2% of children intake them respectively(Table 2).

**Table 2:** Vitamin A rich foods intake among children aged 6–23 months in Ethiopia

S. No	Measurements of food rich in Vitamin A intake	Intake status	
		Count	Percent
1	Eggs	256	18.2
2	Meat (beef, pork, lamb, chicken, etc.)	67	4.8
3	Pumpkin, carrots, squash (yellow or orange inside)	151	10.7
4	Liver, heart, other organs	45	3.2
5	Any dark green leafy vegetables	169	12.0
6	Fish or shellfish	26	1.8
7	Mangoes, papayas, other Vitamin A fruits	162	11.5
Overall vitamin A rich foods among children aged 6–23 months in Ethiopia		549	38.99

### Spatial distribution of vitamin A-rich foods intake among children aged 6–23 months

**Fig. 1:**
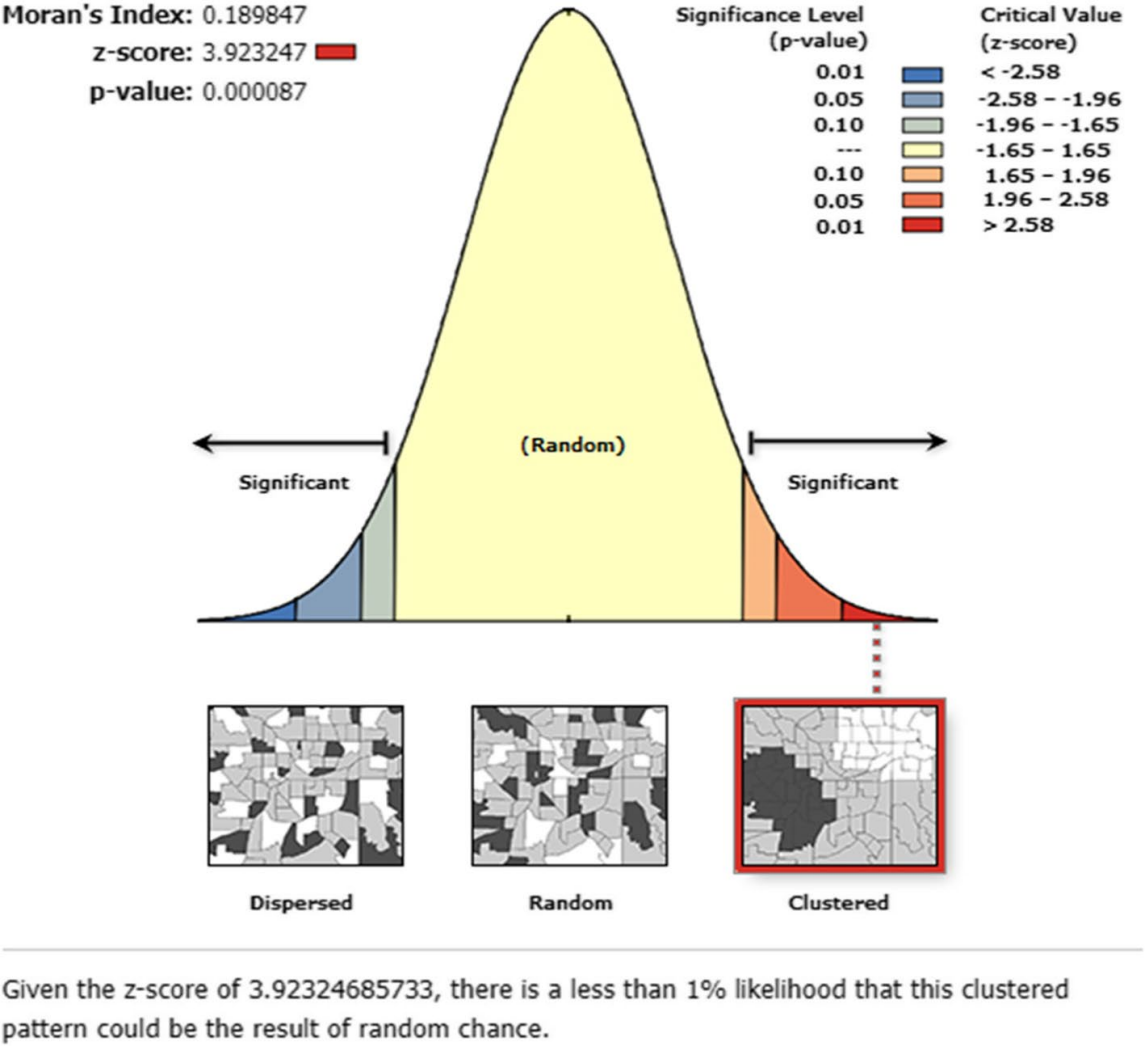
Spatial autocorrelation report of vitamin A rich foods among children aged from 6–23 months in Ethiopia, 2019 EMDHS

The spatial distribution of vitamin A-rich foods intake among children aged 6–23 months was non-random in Ethiopia with (Global Moran’s I = 0.189847, *P*-value = 0.000087)(Fig. 1).

**Fig. 2:**
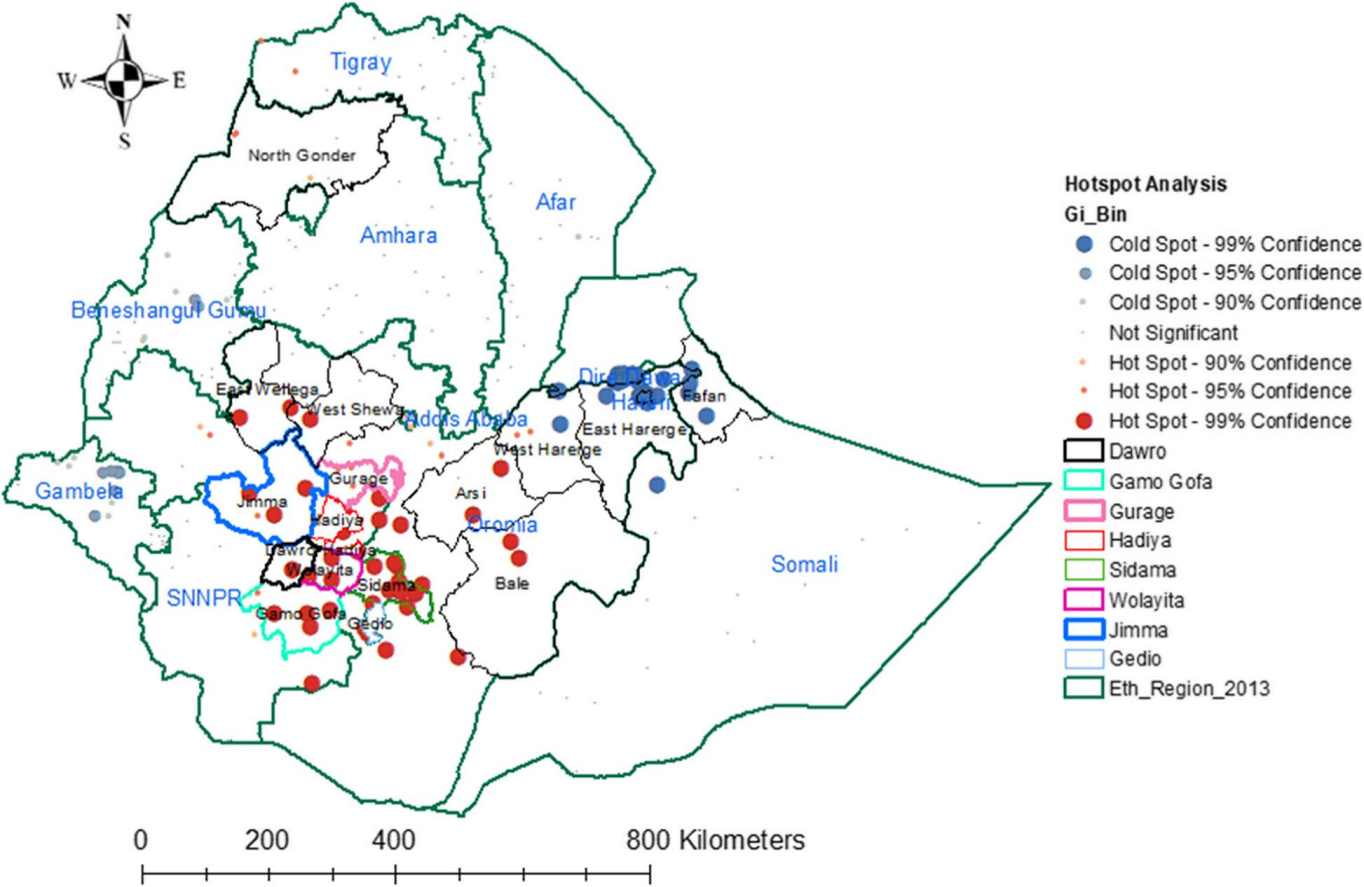
Hot spot analysis for vitamin A rich foods among children aged from 6–23 months, 2019 EMDHS

The high (Hot spots) prevalence of vitamin A rich foods intake among children aged 6–23 months were significantly clustered in SNNPR specifically at Dawro, GamoGofa, Wolayita, Hadiya, Gurage, Sidama Zones, Oromia Region at Jimma, East Wollega, West Shewa and Harerge, Gedio, Arsi and Northern part of Bale Zones, and Amhara Region at North Gondar Zone. Whereas, low (cold spots) intake of foods rich in vitamin A were significantly clustered in Dire Dawa city administration, Harari, Somali (Fafan Zone) Regions of Ethiopia (Fig. 2).

### Spatial SaTScan analysis

**Fig. 3:**
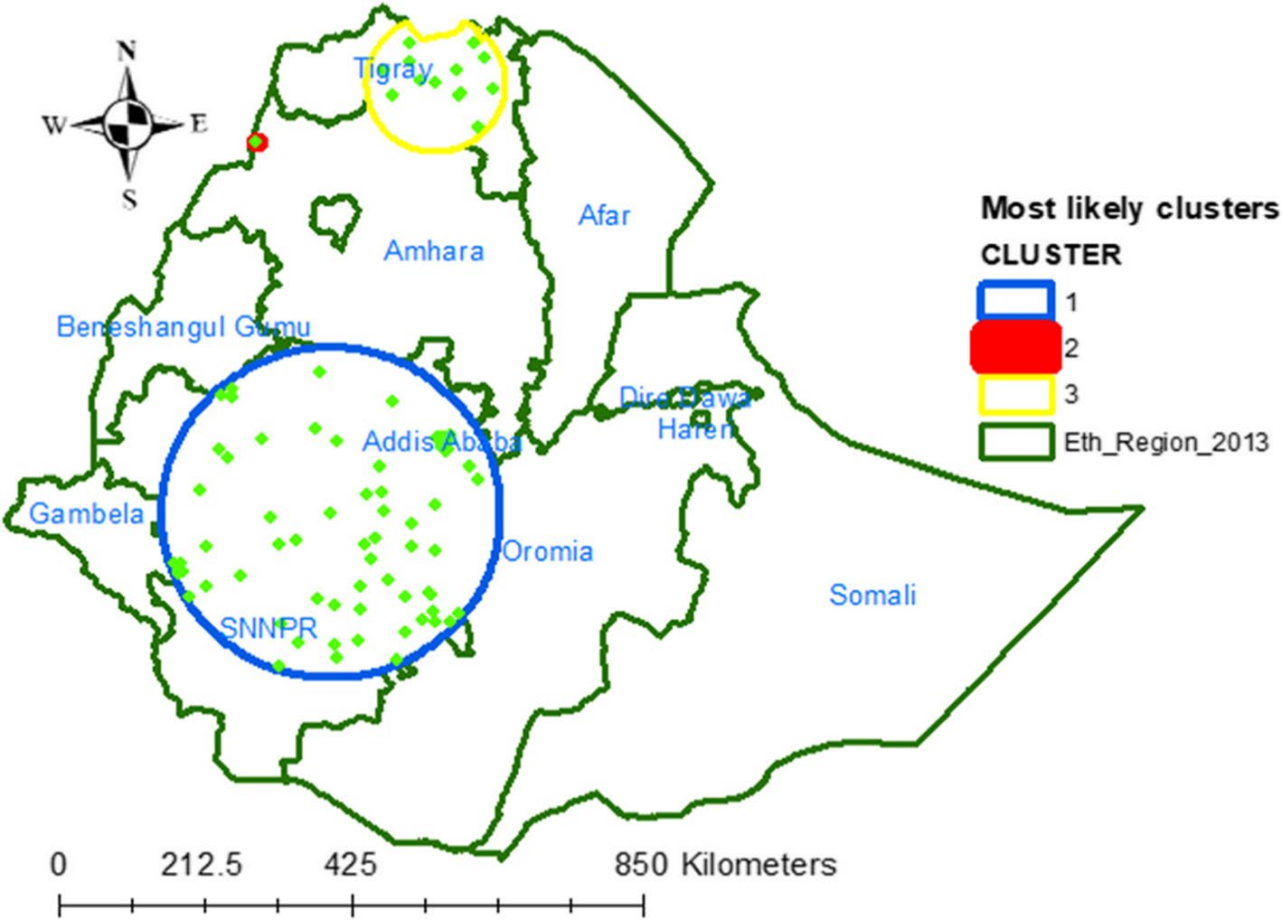
Sat Scan analysis of vitamin A rich foods intake among children aged from 6–23 months

As shown in Fig. 3 below, the blue and red colored windows indicate significant clusters of Vitamin A-rich foods intake among children aged 6–23 months. A total 85 significant clusters were identified. Among 85 significant clusters, 84 were primary and 1 were secondary clusters. The most likely (primary) clusters were located at 8.039877 N, 37.283375 E within a 246.42 km radius in Addis Abeba Oromia and SNNPR Regions of Ethiopia. Children aged 6–23 months who lived in the primary cluster were 70% more likely to intake vitamin A rich foods than children who lived outside the window (RR = 1.70, LLR = 32.38, *P*-value < 0.001). The secondary significant clusters were located at 13.009869 N, 36.258229 E within a 0 km radius in North Gondar Zone of Amhara regional state of Ethiopia (Table 3 and Fig. 3).

**Table 3:** A significant clusters of spatial SatScan analysis for vitamin A rich foods intake among children aged 6–23 months, 2019 EMDHS, in Ethiopia

Tertiary	Secondary	Primary	Types of cluster
10,16,11,13,7, 2, 14, 1, 9, 6, 8, 17, 23, 3	55	95, 174, 91, 179, 177, 176, 171, 96, 97, 180, 112, 87, 189, 205, 98, 203, 204, 178, 191, 93, 190, 175, 195, 116, 184, 120, 196, 187, 274, 99, 92, 159, 194, 260, 278, 261, 277, 185, 275, 262, 279, 276, 94, 280, 270, 263, 258, 257, 197, 264, 265, 198, 273, 267, 256, 266, 173, 271, 272, 268, 269, 119, 181, 115, 201, 101, 199, 90, 186, 168, 223, 221, 222, 167, 169, 227, 224, 172, 183, 226, 117, 200, 192, 104	Detected cluster
(13.810557 N,38.655138E) 101.20 km	(13.009869 N, 36.258229E) 0 km	(8.039877 N, 37.283375E) 246.42 km	Coordinates/ Radius
65	34	581	Populations
38	28	300	Case
1.53	2.16	1.70	RR
5.17	13.72	32.38	LLR
0.481	< 0.001	< 0.001	*p*-value

### Interpolation of vitamin A rich foods among children aged 6–23 months

**Fig. 4:**
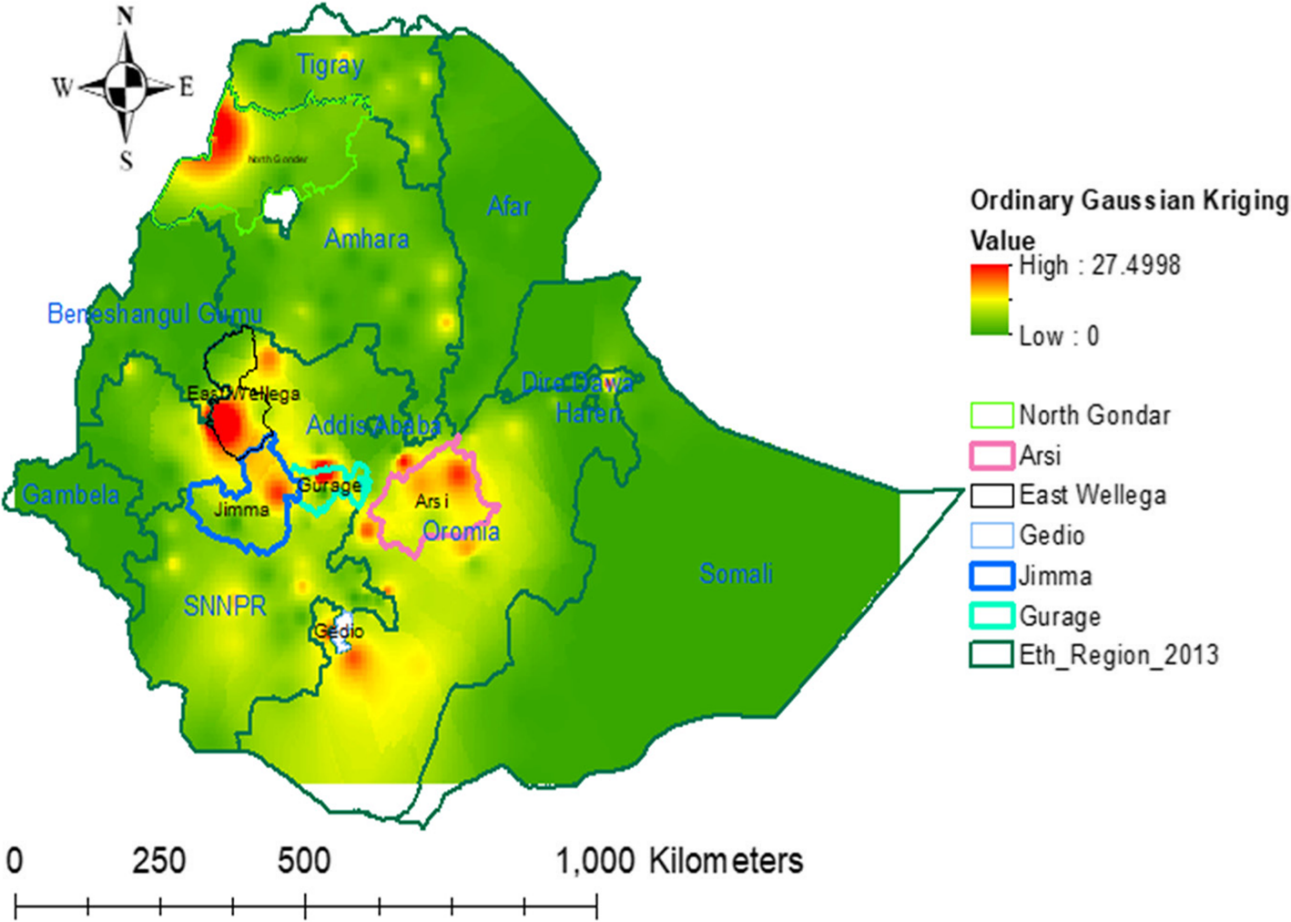
Ordinary Gaussian Kriging interpolation of vitamin A rich foods intake among children aged 6–23 months in Ethiopia

An ordinary Gaussian Kriging interpolation method was employed. The interpolation result indicated that children aged 6–23 months were less likely to intake vitamin A rich foods in most parts of the country. Comparatively, more vitamin A rich foods intake among children aged 6–23 months were observed in Oromia region at Arsi, Gedio, East Wollega, and North East part of Jimma Zones, and Amhara region at North Gondar Zone and SNNPR at Gurage zone (Fig. 4).

### Measure of variation (Random effects)

According to the intraclass correlation coefficient (ICC) and variance report in the empty model, there was 0.302 and 0.284 correlations and variations for vitamin A rich foods intake among children aged 6–23 months. This means that there were 30.1% and 28.4% correlation and total variations in vitamin A rich foods intake among children aged 6–23 months in Ethiopia due to the variations within clusters. Over all model comparisons, and effects of each model (model A,B,C,D) were presented in Table 4.

**Table 4:** Model estimation for vitamin A rich foods intake among children aged 6–23 months in Ethiopia

Measure of variations	Model A	Model B	Model C	Model D
Variance	0.284	0.199	0.153	0.143
ICC	0.302 (.23, .39)	0.188 (.12, .28)	0.124 (.07, .21)	0.096 (.045, .19)
LLR	-862.68	-804.58	-790.01	-758.31
MOR	1.43 (.97, 2.11)	0.76 (.46, 1.27)	0.46(.24, .89)	0.35 (.16, .78)
AIC	1729.37	1643.16	1606.03	1572.63

### Individual and community level factors associated with vitamin A rich foods intake among children aged 6–23 months

In multivariable multilevel mixed effect logistic regression analysis, Educational status of mother, Region, Child age, and Mothers exposure to media were statistically significant factors for vitamin A rich foods intake among children aged 6–23 months in Ethiopia.

After adjusting for other variables, children whose mother is in primary and higher educational status were 1.4 (AOR:1.42, 95% CI: 1.05, 1.93) and 3 (AOR:3.00, 95% CI: 1.59, 5.63) times more likely to intake vitamin A rich foods than children whose mothers had no formal education respectively. Those children whose mother lived in Afar, Amhara, Somali regions and Dire Dawa city were 85% (AOR: 0.15, 95% CI: 0.07, 0.36), 63% (AOR: 0.37, 95% CI: 0.19, 0.71), 99.8% (AOR: 0.02, 95% CI: 0.003, 0.08), and 51% (AOR:0.49, 95% CI: 0.22, 1.12) less likely to intake vitamin A rich foods than children whose mother are from Tigray region respectively. Those children aged 13 to 23 months were 1.8 (AOR: 1.80, 95% CI: 1.38, 2.36) times more odds to intake vitamin A rich foods compared to those children aged 6 to 12 months. Those children whose mother had exposed to media were 1.4 (AOR: 1.41, 95% CI: 1.04, 1.92) times more likely to intake vitamin A rich foods as compared with children whose mother had no media exposure(Table 5).

**Table 5:** Multivariable multilevel mixed effect logistic regression analysis of factors associated with vitamin A rich foods intake among children aged 6–23 months in Ethiopia

Variables	Category	Model A	Model B	Model C	Model D
			AOR (95% CI)	AOR (95% CI)	AOR (95% CI)
Educational status of the mother	Primary		1.58( 1.16, 2.14)^a^	-	1.42(1.05,1.93)^b^
	Secondary		1.54(.93, 2.56)	-	1.52(.93, 2.49)
	Higher		2.74(1.43, 5.22)^a^	-	3.0(1.59,5.63)^b^
	No Formal education		1		1
Religion	Catholic		1.61(.27, 9.69)	-	.85(.56, 4.58)
	Protestant		1.91(1.23, 2.97)^a^	-	1.16(0.7, 1.90)
	Muslim		.84(.59, 1.22)	-	1.40(.90, 2.17)
	Traditional, other		.64(.20, 2.02)	-	.39(.13, 1.21)
	Orthodox		1		1
Wealth	Rich		1.74(1.14, 2.64)^a^	-	1.41(.93, 2.15)
	Middle		1.33(.88, 2.00)	-	1.21(.81, 1.81)
	Poor		1		1
Children’s age (months)	13–23		1.90(1.45, 2.5)^a^	-	1.8(1.38,2.36)^b^
	6–12				1
Media exposure	Yes		1.45(1.06, 1.98)^a^	-	1.41(1.04,1.92)^b^
	No		1		1
ANC visit	Adequate		1.20(.89, 1.62)	-	1.15(.86, 1.54)
	Inadequate		1		1
Place of delivery	Home		.87( .62, 1.20)	-	1.03(.75, 1.42)
	Health facilities		1		1
Baby post-natal check up	Yes		1.13(.76, 1.66)	-	1.05(.72, 1.53)
	No		1		1
Region	Afar		-	.15(.07, .33)^a^	.15(.07, .36)^b^
	Amhara		-	.30(.15, .59)^a^	.37(.19, .71)^b^
	Oromia		-	.78(.41, 1.48)	.73(.36, 1.47)
	Somali		-	.013(.003, .06)^a^	.02(.003, .08)^b^
	Benishangul		-	.89(.45, 1.77)	.95(.56, 1.94)
	SNNPR		-	1.18(.62, 2.23)	1.18(.58, 2.42)
	Gambela		-	1.43(.70, 2.92)	1.58(.72, 3.47)
	Harari		-	.77(.37, 1.60)	.61(.27, 1.36)
	Addis Adaba		-	.76(.34, 1.71)	.55(.24, 1.26)
	Dire Dawa		-	.62(.30, 1.27)	.49(.22, 1.12)^b^
	Tigray			1	1
Residency	Rural	-	-	.47(.31, .70)^a^	.75(.46, 1.21)
	Urban			1	1

^a^Significant at separated model (Model B, C), ^b^Significant at aggregated model (Model D), 1 = Reference category

## Discussion

The present study confirmed that 38.99% (95% CI: 36.46–41.62) of children aged 6–23 months had vitamin A rich foods intake within, or in the last 24 h in Ethiopia. This finding is higher than study done in rural Burundi (16%) and Rwanda (23%) [43], Ethiopia (13.3%-24%) [44]. However, this funding is lower than the reports of study done in different areas such as 58.1% in India [45], 52% in Ghana [19], 79.1% in Malawi [20], and 66.5% in Uganda [21]. Although there is a high (hot spots) for vitamin A rich foods intake among children aged 6–23 months in some parts of SNNPR and Oromia regions of Ethiopia, we can summarize that vitamin A rich foods intake among children aged 6–23 months was not sufficient at national level. This might be high prevalence of poor nutritional status of infants [46, 47], association with receiving appropriate breastfeeding [25, 48], challenging to meet a minimum required food diversity [31]. In addition, insufficient vitamin A rich foods intake among children might be related with mothers’ belief that their children could not chew and digest animal products [46], children might be rarely receiving animal-source foods, the high cost of animal products and using animal product for market purposes [49]. Eggs, any dark fruit leafy vegetables and mangoes, papayas, other Vitamin A fruits were the most intake items of vitamin A rich foods among children aged 6–23 months within, in the last 24 h in Ethiopia. This finding is supported by study done in Ethiopia [50], China [46]. This might be the availability of dense forests and water reservoirs, caregivers that can get wild fruit which are good sources of micronutrients and children might be from households with home garden [31, 51] that enhancing access to fruits and vegetables.

The spatial distribution of vitamin A-rich foods intake among children aged 6–23 months was non-randomly distributed in Ethiopia. A high (Hot spots) vitamin A ich foods intake were observed in Jimma, East Wollega, West Shewa and Harerge, Gedio, Arsi and Northern part of Bale Zones of Oromia Region, North Gondar Zone of Amhara Region, and SNNPR specifically at Dawro, GamoGofa, Wolayita, Hadiya, Gurage, Sidama Zones. Similarly, significant clusters were also detected at Oromia, SNNPR and Amhara Regions of Ethiopia. The spatial Scan statistics report indicate that children aged 6–23 months who lived in the primary cluster were 70% more likely to intake vitamin A rich foods than children who lived outside the window. This might be due to government’s effort to reduce child stunting [52], health extension workers demonstration for mothers about complementary feeding practice and receiving training in the area of nutrition from available nutritional experts [49].

However, Dire Dawa city administration, Harari and Somali (at Fafan Zone) regional states of Ethiopia were vulnerable for high risk of vitamin A rich foods intake among children aged 6–23 months. This might be a delay in receiving and introducing complementary foods at the recommended time [53], low dietary diversity, inconsistency, and less nutritious foods [54]. Sociodemographic characteristics of mothers might be the possible reason [55]. For instance, about 45% of mothers had no formal-education and, majority (83.3% and 75.1%) of households had not Television and radio in this study respectively. This spatial variation might be also the natural variations among individuals, lack of nutritional knowledge, high cost of animal source foods, low household income, low animal production, social norms, and beliefs across the regions of Ethiopia [50].

In multivariable multilevel mixed effect logistic regression analysis, Educational status, Region, Child age, and Media exposure were statistically significant factors for vitamin A rich foods intake among children aged from 6–23 months in Ethiopia.

Children whose mother is in primary and higher educational status were 1.4 (AOR:1.39, 95% CI: 1.05, 1.85) and 3.1 (AOR:3.11, 95% CI: 1.73, 5.59) times more likely to intake vitamin A rich foods than children whose mothers had no formal education respectively. This finding is in line with a similar study conducted in Ethiopia [31, 44, 51, 56, 57], Nepal [58], and India [45]. A possible reason might be more educated women have good skills to access modern health services and more likely to understand messages about dietary diversity, health [43], and previous experience about minimum dietary diversity [59, 60]. Plus, mothers who attend formal education might have a better know how about better child feeding, linkage between maternal knowledge and the quality of diet offered to their children [31], and higher educational status and over all literacy of mother influence children to meet infant feeding guidelines [61] might be a reason for this finding. Lastly, unable to access of information due to poor education most often connected to poor supplementation of vitamin A [56]. In this study, 45% of children’s where from mother who had no formal education.

Those children whose mother lived in Afar, Amhara, Somali and Dire Dawa were 85%, 63%, 99.8%, and 51% less likely to intake vitamin A rich foods. This evidence is supported by a reports that Afar, Amhara, Somali regions and Dire Dawa city are negatively associated with adequate dietary diversity [44], pastoralist (Afar and Somali) and Amhara regions are had faced problems for high risk of vitamin A supplementation [56]. This might be due to these regions might be vulnerable for anthropometric failures [57, 62], poor resource distribution, less access to services [56], and scarcity of vitamin A rich foods [8].

Those children aged 13 to 23 months were 1.8 (AOR: 1.80, 95% CI: 1.38, 2.36) times more odds to intake vitamin A rich foods compared with children aged 6 to 12 months. This evidence is in line with local studies done about the feeding practices, and stunting of children [57, 63], further analysis of 2016 EDHS in the emerging regions of Ethiopia [8]. This might be the amount of food consumed per day increased as the age of the children progressed [54], older age groups children probably have better dietary diversity as they could eat family meals for themselves, and children above 12 months old were more likely to obtain diversified food [64, 65]. Plus, mother’s poor perceptions and traditional belief’s contribute to low intake vitamin A rich foods among those children aged 6–12 months [8], the associations of younger (6–12 months) children with inadequate dietary diversity, and delay in imitation of complementary feeding in the form of solid, semi-solid food [66–69] might be a reason for children aged 6–12 months for less likely to intake vitamin A rich foods than 13–23 months old children in this study.

In the present study, media significantly affects the intake of vitamin A rich foods among children aged from 6-to 23 months in Ethiopia. Those children whose mothers had media exposure were 1.4 (AOR: 1.41, 95% CI: 1.04, 1.92) times more likely to intake vitamin A rich foods than children whose mother had no media exposure. This finding is in line with studies done in different parts of Ethiopia [31, 51, 56, 57, 65, 70], Bangladesh [71], and Indonesia [72]. This might be the impact of media for promoting child feeding practice and mothers’ exposure to media able to feed different foods groups to their children.

## Conclusion

Vitamin A rich foods intake among children aged 6–23 months across the region of Ethiopia is insufficient at national level. The spatial distributions are non-random, and the cold spots (low prevalence) are being observed in the Fafan zone of Somali and Harari regions, and Dire Dawa city administration of Ethiopia. Independent variables such as Educational status of c mother, Region, Child’s age, and Media exposure were significantly associated with vitamin A rich foods intake among children aged 6–23 months. Researchers needed to recommend stakeholders to strengthen educational status of mother, creating awareness of mother on child feeding, delivering nutritional related message using media, and using locally available natural resource for the productions of vitamin A rich foods may be good for further enhancement of vitamin A rich foods intake among children aged 6–23 months.

### Strength and limitations of the study

Since the study is done through using EMDHS national datasets which the finding may have good generalizability. Conducting multilevel mixed effect model to alleviate the effects of cluster is considered as the strength of this study.

As a limitation, since the data was collected retrospectively it may prone recall bias. As long as the 2019 EMDHS dataset has no observation for some variables, important variables which determine vitamin A rich foods intake among children may not be included under this study.

## Data Availability

The dataset used for analysis is available on the Measure DHS program (http://dhsprogram.com) website. All the data generated and analyzed during this study are included, in the form of maps, tables, and texts, in this article.

## Abbreviations

AIC: Akaike's Information Criteria
ANC: Antenatal Care
AOR: Adjusted Odds Ratio
CI: Confidence Interval
CSA: Central Statistical Agency
COR: Crude Odds Ratio
DHS: Demographic and Health Survey
EAs: Enumeration Areas
EDHS: Ethiopian Demographic and Health Survey
EMDHS: Ethiopia Mini Demography and Health Survey
EPHC: Ethiopian Population and Housing Census
EPHI: Ethiopian Public Health Institute
ICC: Intraclass Correlation Cofficient
RR: Relative Risk
LLR: Log likelihood Ratio
MOR: Median Odds Ratio
MNs: Micronutrients
SNNPR: South Nations Nationalities and People’s Region
STATA: Statistical Software for Data Science
VAD: Vitamin A Deficiency
WHO: World Health Organization
